# A dual-axis cisternal classification for congenital intracranial cystic lesions: implications for surgical strategy and long-term prognosis

**DOI:** 10.1007/s00701-025-06722-1

**Published:** 2025-12-01

**Authors:** Maria Mihaela Pop, Dragos Bouros, Artsiom Klimko, Ioan Alexandru Florian, Cristian Ionel Abrudan, Ioan Stefan Florian

**Affiliations:** 1 Department of Neurosurgery, Iuliu Hatieganu University of Medicine and Pharmacy, Cluj-Napoca, Romania; 2 Clinic of Neurosurgery, Cluj County Emergency Clinical Hospital, Cluj-Napoca, Romania; 3 Department of Neurosurgery, University Hospital Balgrist, Zürich, Switzerland; 4 Department of Maxillofacial Surgery and Radiology, Iuliu Hatieganu University of Medicine and Pharmacy, Cluj-Napoca, Romania

**Keywords:** Congenital intracranial cystic lesions, Cisternal anatomy, Surgical outcomes, Recurrence, Topographic classification

## Abstract

**Background:**

Congenital intracranial cystic lesions—epidermoid, dermoid, neurenteric, Rathke, colloid—encompass a heterogeneous group of entities whose surgical behavior is only partly explained by histology. We propose a dual-axis cisternal topographic model (medial–lateral × dorsal–ventral) to compare surgical outcome in congenital intracranial cystic lesions.

**Methods:**

We retrospectively analyzed 110 patients with histologically confirmed congenital intracranial cysts undergoing surgical resection at a single tertiary center. Each lesion was categorized by cisternal topography in coronal and axial planes, and correlations were assessed between topographic class, surgical parameters, recurrence, and outcomes. Inter-rater reliability of classification was measured using Cohen’s kappa.

**Results:**

Lesions with complex cisternal topography—defined as extension across dorsal–ventral (multicompartmental, MCL) or combined median-paramedian compartments—showed higher recurrence (42.1% and 50%, respectively; *p* < 0.001), including late recurrence beyond five years (23.7% and 35%, respectively; *p* < 0.001). MCL carried greater surgical morbidity: higher rates of subtotal resection (44.7% vs. 8.3%, *p* < 0.001), prolonged hospitalization, and doubled complication burden (52.6% vs. 25%, *p* = 0.006). Functional outcomes were poorer in these subgroups (median GOS = 4, IQR: 4–5), and neurological sequelae, particularly cranial nerve VII/VIII deficits and cerebellar signs, were disproportionately more frequent. A strong correlation emerged between cyst topography and histopathology, epidermoids predominating in complex configurations (*p* < 0.001). Supplementary analyses indicated that although cyst size varied across histological subtypes, it was not associated with recurrence, and in multivariable models cisternal localization remained significant, whereas histology and maximum dimension did not.

**Conclusions:**

Cisternal topography was associated with recurrence, surgical complexity, and postoperative outcome in congenital cystic lesions. Lesions with multicompartmental or midline-paramedian axial spread carry a high-risk profile and warrant extended surveillance beyond five years. This dual-axis anatomical model may inform more tailored operative strategies and long-term follow-up planning, complementing histological diagnosis in modern skull base surgery.

**Supplementary Information:**

The online version contains supplementary material available at 10.1007/s00701-025-06722-1.

## Introduction

Congenital intracranial cyst—including epidermoid, dermoid, neurenteric, Rathke’s cleft, and colloid types -arise from aberrant embryologic development and, although rare, collectively pose considerable neurosurgical challenges. Their unpredictable growth, varied cisternal locations, and propensity for neurovascular compression or cerebrospinal fluid (CSF) obstruction complicated management. While histopathology is the diagnostic gold standard, it offers limited guidance for surgical decision-making in deep-seated anatomically complex lesions [1, 4, 6, 8, 9, 11, 13, 14, 16, 20, 22, 28, 29].

In recent years, renewed attention to the brain’s cisternal anatomy has underscored its value as a surgical roadmap. Subarachnoid cisterns—bounded by neurovascular, bony, and dural structures—define accessibility, risk profile, and resectability. Yet, most studies still evaluate congenital cystic lesions through a histopathologic lens, without integrating detailed anatomical topography as a prognostic factor [2, 5, 9, 15, 21, 23, 25–27].

To address this gap, we present and prospectively applied a dual-axis cisternal classification model based on two orthogonal anatomical dimensions: medial–lateral and dorsal–ventral. This framework translates radiological topography into a pre-operative tool for planning and outcome prediction. We hypothesize that cisternal localization is independently associated with surgical complexity, extent of resection, and recurrence—and may outperform histological subtype in predicting clinical course.

In this study, we analyze a single-center cohort of 110 histologically confirmed congenital cystic lesions, captured through a hybrid restrospective-prospective design, to test the clinical utility of our topographic model. By quantitatively correlating cisternal location with recurrence and morbidity data, we aim to develop a topography-informed risk stratification model that may augment traditional histological classification and improve surgical decision-making, patient counseling, and follow-up protocols.

## Methods

### Study design and patient selection

We conducted a single-center, observational cohort study conducted at the Department of Neurosurgery, Cluj-Napoca County Emergency Clinical Hospital—North-Western Romania’s tertiary neurosurgical referral unit. All patients with histologically confirmed congenital intracranial cystic lesions who underwent surgery between 2008 and 2024 were eligible. This cohort incorporates and expands upon our previously reported institutional series, including the 36 cases of intracranial epidermoid cysts published separately, as well as 7 cases included in a prior systematic review. These patients were re-analyzed within the present framework together with newly accrued cases, thereby ensuring a uniform application of the dual-axis cisternal classification. Institutional review board approval was obtained, and prospective inclusions (2021 onward) provided written informed consent. The study conforms to the Declaration of Helsinki and Romanian College of Physicians regulations.

A hybrid design was applied: cases from 2008 and 2020 were identified retrospectively via electronic records and operative reports, whereas cases from 2021–2024 were captured prospectively using a uniform data-collection template. This approach ensured consistent longitudinal documentation across the 16-year period.

### Inclusion and exclusion criteria

Inclusion criteria were:

- Histologically proven congenital intracranial cystic lesion (e.g., epidermoid, dermoid, neurenteric, Rathke’s cleft, colloid cysts);
- Availability of complete pre-operativ Magnetic Resonance Imaging (MRI) permitting cisternal classification;
- A minimum postoperative follow-up of 12 months, including at least three documented clinical and/or radiological evaluations to assess recurrence and functional outcome;
- Undergoing neurosurgical resection with curative intent.

Exclusion criteria included acquired cysts (post-traumatic, infectious, or neoplastic origin), cystic tumors with solid components, and incomplete radiological or histological documentation.

### Data collection and classification parameters

Demographics, clinical, radiological, surgical, and histopathological variables were extracted. Pre-operative MRIs (with or without contrast) were independently reviewed by two senior neurosurgeons; disagreements were resolved by consensus with a neuroradiologist. Lesions were assigned to a dual-axis cisternal classification system that incorporated both coronal and axial neuroanatomical dimensions. In the axial plane, cisternal compartments were classified along a medial–lateral (median-paramedian) continuum relative to the midline structures of the brainstem. Cisterns situated strictly along the midline—such as the interpeduncular, prepontine, medullary (anterior), suprasellar, chiasmatic, quadrigeminal, and pericallosal—were designated as *median* due to their alignment with the anatomical midline and their embryologic derivation from central neuroectodermal structures. In contrast, *paramedian cisterns* included those that lie lateral but adjacent to the midline, such as the crural, ambient, sylvian, and superior cerebellar cisterns, which are often accessed through lateral surgical corridors and demonstrate asymmetric extension. Lesions spanning both medial and paramedian compartments, or crossing the anatomical midline, were classified as *multicompartmental*. In the coronal plane, *ventral* localization was defined relative to the anterior surface of the brainstem, rather than skull base anatomy. Lesions were classified as ventral if the majority of their volume was situated anterior to the midbrain or pons, typically occupying the interpeduncular, prepontine, medullary, sylvian, chiasmatic, suprasellar, or crural cisterns. Conversely, *dorsal* lesions were defined as those located posterior or superior to the brainstem, involving compartments such as the quadrigeminal, ambient, pericallosal, and superior cerebellar cisterns. Lesions were labeled *multicompartmental (complex)* if they demonstrated extension across both dorsal and ventral planes or involved cisternal junctions with intercompartmental communication. Strictly non-cisternal midline cysts (e.g., purely intrasellar or intraventricular) were classified separately. Inter-rater agreement was quantified using Cohen’s kappa (κ) statistics, with values > 0.80 considered excellent.

Representative preoperative MRI characteristics of each topographic subgroup are illustrated in Table 1, highlighting the anatomical distribution, imaging features, and cisternal compartmentalization relevant to surgical planning.

**Table 1:**
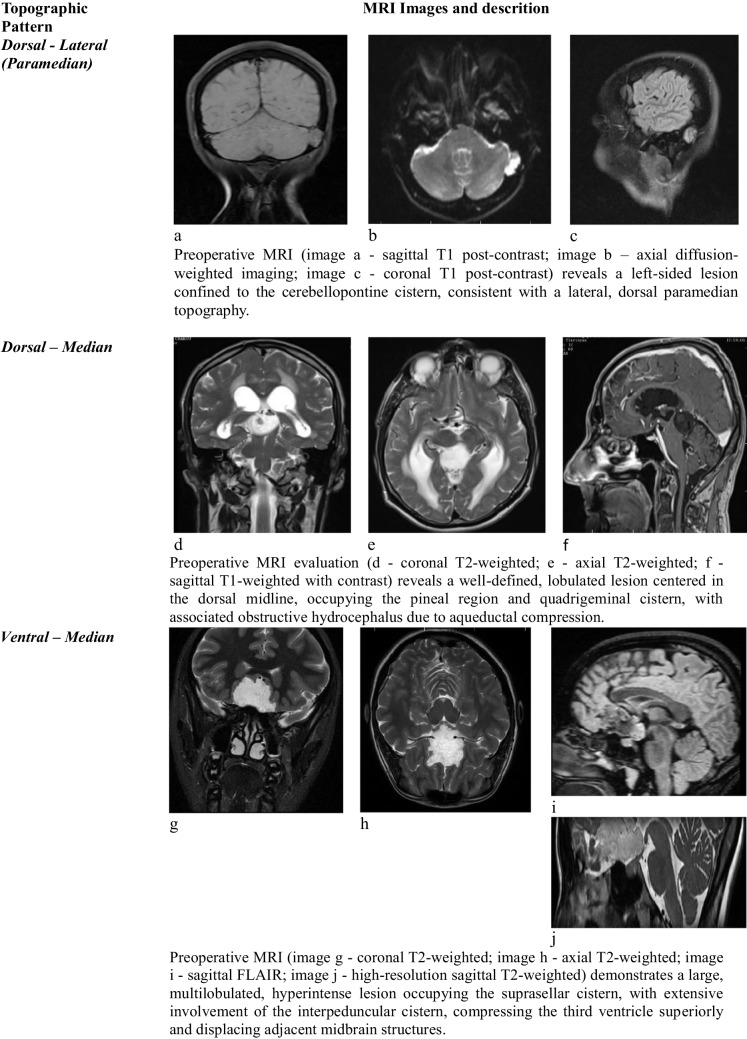

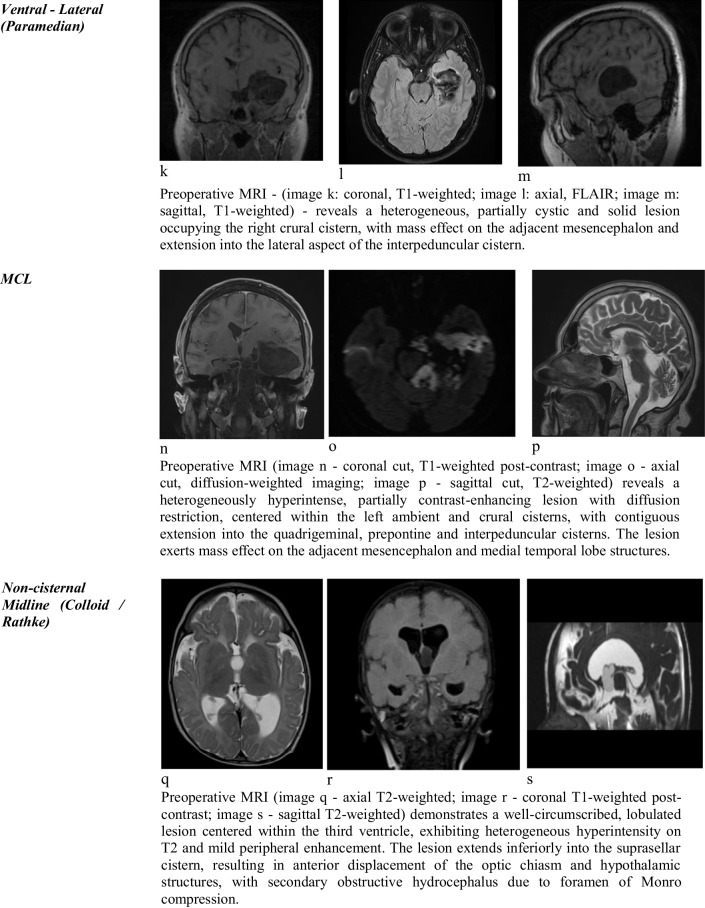
Topographic classification of intracranial cystic lesions with representative MRI sequences

All patients underwent microscopic surgical resection. Gross total resection (GTR) was defined as complete removal of both cyst contents and capsule, with no residual lesion on the 3-month postoperative MRI. Subtotal resection (STR) was defined either as residual capsule left behind despite full cyst evacuation or partial removal of both contents and capsule. The extent of resection was determined through intraoperative documentation and verified by early postoperative imaging. All patients received a postoperative non-contrast CT scan (Computerized Tomography scan) within 24 h to evaluate for hemorrhage or other immediate surgical complications. Follow-up evaluations were conducted in the outpatient neurosurgery clinic at standardized intervals.

Radiological follow-up included pre-operative imaging (MRI mandatory; CT when available), immediate postoperative MRI, and scheduled follow-up MRI at 3 months, 6 months, 1 year, and annually thereafter. Recurrence was defined as any increase in residual cyst volume or a new cystic focus at the surgical site. Hydrocephalus at presentation and the need for CSF diversion (External Ventricular Drain (EVD) or Ventriculo-Peritoneal Shunt (VPS)) were also recorded.

Post-operative data captured early complications (neurological, infectious, or systemic), length of stay, intensive-care requirement, and functional outcome (Glasgow Outcome Scale—GOS) at discharge and last follow-up. Neurological deficits were by system (cranial nerve (CN), motor/sensory, cerebellar) and timing (pre-existing versus new/worsened deficits).

### Statistical analysis

Statistical analyses were performed using IBM SPSS v25. Categorical variables presented as counts (%) and compared using Pearson χ^2^ or Fisher’s Exact test; Bonferroni-adjusted pairwise Z-tests followed significant omnibus results. Continuous variables are reported as means with standard or medians with interquartile ranges depending on Shapiro–Wilk normality testing; comparisons employed independent t-tests (equal variances verified with Levene) or Mann–Whitney U as appropriate.

Associations between cisternal localization and surgical endpoint (subtotal resection, complications, recurrence) were evaluated with univariable and multivariable binomial logistic regression. Odds ratios (ORs) with 95% Confidence Intervals (CIs) are reported. Statistical significance was defined as two-tailed α = 0.05.

Classification reproducibility was interpreted according to Landis and Koch benchmarks.

## Results

### Cohort characteristics and outcomes

A total of 110 patients met inclusions criteria—their median age at surgery was 42 [Interquartile Range (IQR): 26.7–53.2] and 50.9% were female. Age distribution peaked in the fifth and sixth decades (51–60 years: 24.5%) and remained substantial in the 21–40-year group (31%), so that 52.7% of the cohort was older than 40 at diagnosis. Symptom onset was predominantly insidious (80.6%), with half presenting between 1 month and 1 year.Median hospital stay was 9 days [IQR: 8–12.5], with 40.4% exceeding 10 days.

Most lesions were supratentorial (57.3%). Histopathology showed a predominance of epidermoid cysts (51.8%), with colloid (23.6%), Rathke cleft (16.4%), dermoid (4.5%) and neurenteric cysts (3.6%) less frequent. Surgical strategies achieved total resection in 79% of cases. Recurrence was identified in 20.9% (*n* = 23), with a mean recurrence interval of 5.91 ± 4.32 years; notably, 9.1% recurred beyond five years of follow-up.Overall, 34.5% of patients experienced at least one post-operative complications. To ensure transparent reporting and traceability, we compiled a master table summarizing the distribution of histological subtypes, cisternal localization, extent of resection, and major outcomes across the overall cohort (Table 2).

**Table 2:** Traceable data inventory: Histology, cisternal topography, resection, and outcomes

Block	Item	Overall count
Histology	Epidermoid	57
	Dermoid	5
	Neurenteric	4
	Rathke’s cleft	18
	Colloid	26
Cisternal localization	Axial median–paramedian	20
	Supratentorial	63
	Infratentorial	28
	Transtentorial	19
Extent of resection	GTR	87
	STR/fenestration	23
Outcomes (overall)	Any postoperative complication	38
	-New/worse cranial-nerve deficit	6
	-Pseudomeningocele	2
	-Transient hydrocephalus (with SDHyg)	1
	-Diabetes insipidus	3
	-Subdural hygroma—isolated	5
	-Acute subdural hematoma with seizure	1
	-Pulmonary embolism	1
	-Mesenteric lymphadenitis	1
	-Dysartria	1
	-Cardiorespiratory arest	1
	-CSF diversion—EVD	16
	-CSF diversion—VPS	9
	Reoperation ≤ 30 days	8
	Reoperation (late)	20
	Recurrence (any during FU)	23
	Late recurrence > 5 years	11 (*n*, not %)

### Inter-rater reliability of cisternal classification

For the coronal axis location, Cohen’s kappa = 0.935 (Standard error = 0.028), for the axial axis location, Cohen’s kappa = 0.950 (Standard error = 0.024). Both indicate almost perfect agreement between examiners.

### Coronal cisternal classification and surgical complexity

Stratification along the coronal (dorsal–ventral) axis, according to Liliequist line, revealed that 38 patients (34.5%) exhibited complex cisternal topography, defined by multicompartmental extension across dorsal and ventral cisternal planes.

Histopathology, epidermoid cysts predominated (51.8%),being significantly more frequent in multicompartmental locations (84.2% vs. 34.7%) whereas colloid cysts (36.1% vs. 0%) and Rathke cleft cysts (25% vs. 0%) were confined to localized compartments (*p* < 0.001).

Sulgically, multicompartmental lesions (MCL) Showed higher rates of subtotal resection (44.7% vs. 8.3%, *p* < 0.001), more frequent advanced approaches (retrosigmoid (47.4%) and median suboccipital (28.9%) craniotomies, < 0.001), and predominant use of the sitting position (63.2% vs. 12.5%, *p* < 0.001).

MCL were also associated with prolonged hospitalization (median: 10 vs. 9 days), increased recurrence (42.1% vs. 9.7%, *p* < 0.001), and a significantly elevated recurrence risk (OR: 6.75; 95% CI: 2.45–18.56). Recurrences occurring beyond 5 years were disproportionately more frequent in this group (23.7% vs. 2.8%, *p* < 0.001) compared to localized cisterns. They also correlated with infratentorial and transtentorial locations, greater need for cerebrospinal fluid diversion and nearly double the rate of postoperative complications. Clinically, CN VII palsy and cerebellar signs were more frequent, and functional outcomes were poorer compared to localized lesions (median GOS 4 vs. 5, *p* = 0.025). A full overview is providen in Tables 3 and 4.

**Table 3:** Baseline characteristics, imaging/pathology, and outcomes stratified by cisternal complexity (Complex vs. Simple Lesions)

Category/Variable	Complex (MCL)	Simple (Confined)
Baseline characteristics		
Group size (*n*)	38	72
Age, years—median (IQR)	43 (27.5–53.2)	41.5 (26.2–53.7)
Sex—female, *n*	23	33
Pre-op CN deficit present, *n*	14	18
Hydrocephalus at presentation, n	7	23
Follow-up, months—median (IQR)	3 (1–5.25)	2 (1–4)
Imaging & pathology		
Axial median–paramedian involvement, *n*	18	2
Supratentorial location, *n*	4	59
Infratentorial location, *n*	18	10
Transtentorial location, *n*	16	3
Histology—Epidermoid, *n*	32	25
Histology—Dermoid, *n*	3	2
Histology—Neurenteric, *n*	3	1
Histology—Rathke’s cleft, *n*	0	18
Histology—Colloid, *n*	0	26
Outcomes		
Extent of resection—GTR, *n*	21	66
Extent of resection—STR/fenestration, *n*	17	6
Postoperative complication, *n*	20	18
CSF diversion—EVD, *n*	0	16
CSF diversion—VPS, *n*	6	3
Reoperation ≤ 30 days, *n*	1	7
Reoperation (late), *n*	15	5
Recurrence (any during FU), *n*	16	7
Late recurrence > 5 years, *n*	9	2

**Table 4:** Correlation between the cyst location according to classification on CORONAL axis and different variables

Variables	Localized/Complex Cisterns (p*)
Neurological signs (CN VII palsy)	0.017
Neurological signs (Cerebellar signs)	0.029
Recurrence (Absent, Present)	< 0.001
Recurrence interval (No recurrence, 1–5 years, > 5 years)	< 0.001
Cyst location (Supratentorial, Infratentorial, Transtentorial)	< 0.001
Degree of removal (Subtotal, Total)	< 0.001
Surgical approach (Retrosigmoid, Telovelar, Other)	< 0.001
Histopathology (Colloid cyst, Dermoid cyst, Epidermoid cyst, Neurenteric cyst, Rathke cleft cyst)	< 0.001
Postoperative complications (Absent, Present)	0.006
CSF diversion procedures (Absent, EVD, VPS)	< 0.001
GOS (Median (IQR))	0.025

### Axial cisternal topography and its relationship to clinical and surgical outcomes

Analysis in the axial (medial–lateral) plane identified 20 patients (18.2%) whose cysts spanned both median and paramedian cisterns (Table 5 and 6). These lesions constituted the most aggressive subgroup: recurrence reached 50% versus 14.4% for laterally confined cysts (*p* = 0.001), giving an odds ratio of 5.92 (95% CI: 2.06–17.01). In 35% of cases recurrence emerged beyond five years (*p* < 0.001), underscoring the need for extended surveillance.

**Table 5:** Baseline characteristics, imaging/pathology, and outcomes stratified by cisternal complexity (Median & Paramedian vs. Simple Lesions)

Category/Variable	Median & Paramedian	Simple (Confined)
Baseline characteristics		
Group size (*n*)	20	90
Age, years—median (IQR)	47 (31–53.75)	41.5 (25.75–53.25)
Sex—female, *n*	11	45
Pre-op CN deficit present, *n*	8	24
Hydrocephalus at presentation, *n*	4	26
Follow-up, months—median (IQR)	3 (1.25–7.5)	2.5 (1–4)
Imaging & pathology		
Supratentorial location, *n*	2	61
Infratentorial location, *n*	6	22
Transtentorial location, *n*	12	7
Histology—Epidermoid, *n*	18	39
Histology—Dermoid, *n*	2	3
Histology—Neurenteric, *n*	0	4
Histology—Rathke’s cleft, *n*	0	18
Histology—Colloid, *n*	0	26
Outcomes		
Extent of resection—GTR, *n*	6	81
Extent of resection—STR/fenestration, *n*	14	9
Postoperative complication, *n*	9	29
CSF diversion—EVD, *n*	0	16
CSF diversion—VPS, *n*	3	6
Reoperation ≤ 30 days, *n*	1	7
Reoperation (late), *n*	9	11
Recurrence (any during FU), *n*	9	9
Late recurrence > 5 years, *n*	7	3

**Table 6:** Correlation between the cyst location according to classification on AXIAL axis and different variables

Variables	Localized/Median & Paramedian Cisterns (p*)
Neurological signs (Sphincter disturbances)	0.020
Neurological signs (CN VII palsy)	0.004
Neurological signs (CN VIII Hearing difficulty)	0.004
Neurological signs (Cerebellar signs)	0.013
Recurrence (Absent, Present)	0.001
Recurrence interval (No recurrence, 1–5 years, > 5 years)	< 0.001
Cyst location (Supratentorial, Infratentorial, Transtentorial)	< 0.001
Degree of removal (Subtotal, Total)	< 0.001
Surgical approach (Retrosigmoid, Telovelar, Other)	< 0.001
Histopathology (Colloid cyst, Dermoid cyst, Epidermoid cyst, Neurenteric cyst, Rathke cleft cyst)	< 0.001
CSF diversion procedures (Absent, EVD, VPS)	0.037
GOS (Median (IQR))	0.001

Anatomically, 60% of these lesions were transtentorial (*p* < 0.001), and 70% underwent only subtotal resection, typically via retrosigmoid craniotomy (75% vs. 16.7%, *p* < 0.001). Outcomes were poorer, with a lower median GOS (median = 4, IQR = 4–5 vs. median = 5, IQR = 4–5, *p* = 0.001), increased surgical burden (median number of operations: 1.5 vs. 1, *p* = 0.009), and high rates of neurological sequelae, including sphincter disturbance (20% vs. 3.3%, *p* = 0.020), cranial nerve VII palsy (35% vs. 7.8%, *p* = 0.004), cranial nerve VIII hearing loss (35% vs. 7.8%, *p* = 0.004), and cerebellar signs (30% vs. 7.8%, *p* = 0.013). Histologically, median and paramedian lesions were overwhelmingly epidermoid (90% vs. 0%, *p* < 0.001).

### Impact of cyst size on recurrence

To further examine whether cyst size acted as a confounding factor, maximum dimensions were analyzed across histological subtypes and recurrence groups.

**Fig. 1:**
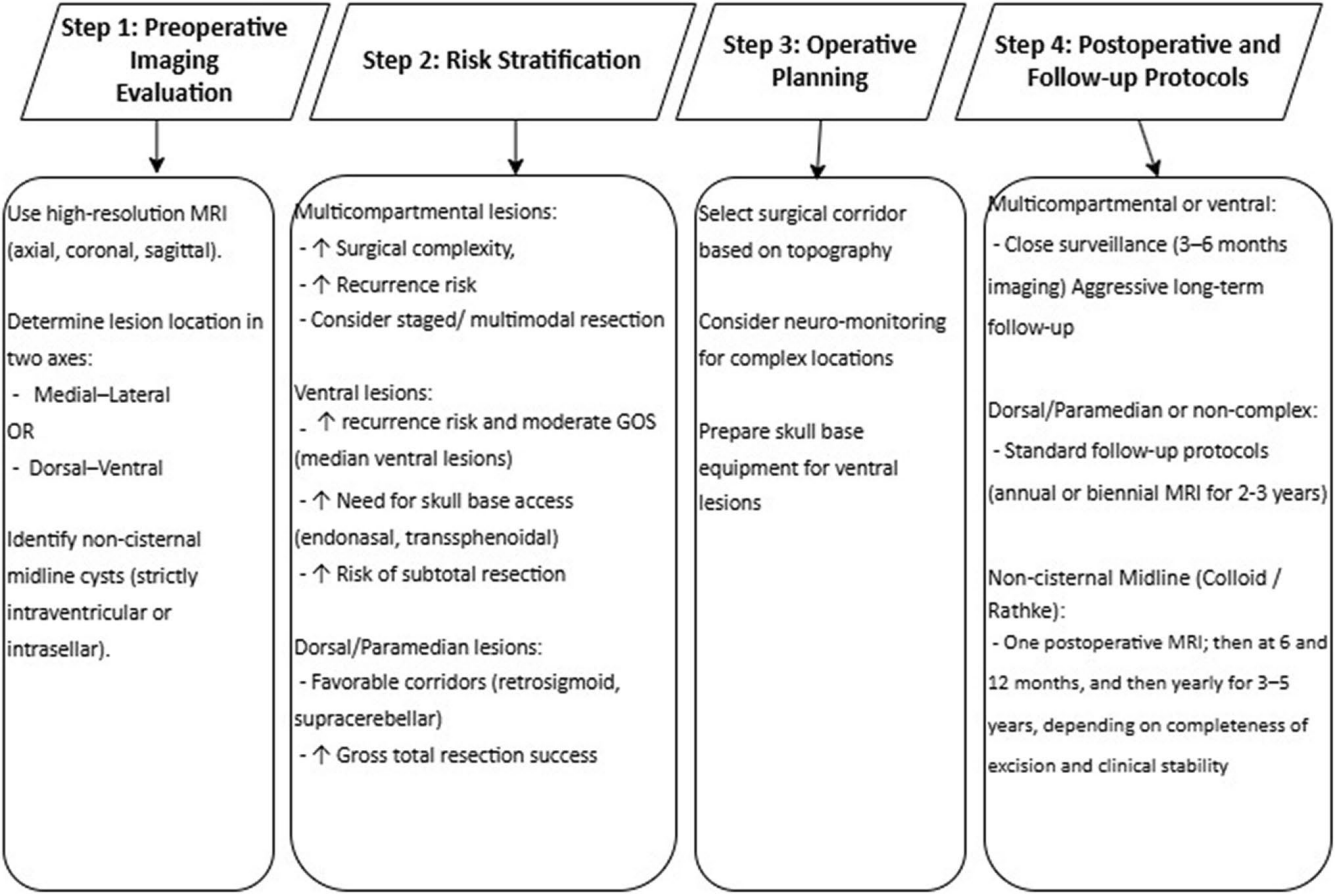
Surgical algorithm based on cisternal topographic classification

*Comparison by histology*. Maximum linear measurements along the anteroposterior, laterolateral, and craniocaudal axes differed significantly among histological types (Supplementary Table 1, Fig. 1). d cysts were significantly larger than colloid cysts (*p* = 0.002) and Rathke’s cleft cysts (*p* < 0.001), while neurenteric cysts demonstrated the greatest craniocaudal extension. These findings confirm that cyst size varies systematically with histological type and may influence operative complexity.

*Comparison by recurrence*. When patients were stratified according to the presence or absence of recurrence, no significant differences in cyst dimensions were identified (AP, *p* = 0.087; LL, *p* = 0.248; CC, *p* = 0.154) (Supplementary Table 2, Fig. 2).

*Multivariable analyses*. To further evaluate this potential confounder, maximum anteroposterior dimension was entered as a covariate in logistic regression models. Cisternal localization on both the coronal and axial axes remained a significant and independent potential predictor of recurrence (*p* = 0.001 and *p* = 0.006, respectively), whereas maximum dimension itself had no measurable effect (Supplementary Table 3).

Taken together, these results suggest that although cyst size varies by histological type, it does not independently influence recurrence.

### Relative prognostic contribution of histology vs. Cisternal localization

Histopathological subtypes showed significant clustering within specific cisternal configurations: epidermoid cysts predominated in complex coronal extensions (84.2% vs. 34.7%, *p* < 0.001) and in median/paramedian axial locations (90% vs. 43.3%, *p* < 0.001), while colloid and Rathke’s cleft cysts were markedly underrepresented in these sites (*p* < 0.001). In multivariable regression models including both histology and cisternal localization, only localization retained independent prognostic value. Complex coronal extension (OR = 8.09, 95% CI 2.39–27.37, *p* = 0.001) and median/paramedian axial involvement (OR = 5.62, 95% CI 1.76–17.99, *p* = 0.004) were potential predictors of recurrence, whereas histological subtype did not reach statistical significance.

### Patern of recurrence across topographic strata

Cisternal topography stratified recurrence in both planes. In the coronal axis, recurrence rates differed markedly across topographic subgroups (*p* < 0.001), with post hoc Z-tests (Bonferroni corrected) indicating that MCL were significantly more likely to recur (69.6% of recurrence cases vs. 25.3% of non-recurrence cases). Similarly, recurrence risk differed significantly by axial localization (*p* < 0.001). Median and combined median-paramedian cisternal involvement were strongly associated with recurrence (39.1% and 43.5% among recurrence cases, respectively), whereas paramedian (16.1% vs. 0%) were associated with reduced recurrence likelihood. A subset of patients (*n* = 18, 16.4%) exhibited both multicompartmental (coronal) and combined median-paramedian (axial) extension. This subgroup had a markedly higher recurrence rate (55.6% vs. 14.1%, *p* < 0.001, OR 7.60, 95% CI: 2.53–22.80).

## Discussion

This study presents an anatomical and clinical analysis of congenital intracranial cysts, framed by a dual-axis cisternal classification. By considering medial–lateral and dorsal–ventral orientation simultaneously, we observed that cisternal topography, rather than histological subtype or cyst size, aligned most closely with subtotal resection, complications, and recurrence. Lesions extending across multiple cisternal compartments or situated at the midline-paramedian axis were consistently more difficult to resect completely, carried a higher burden of complications, and showed increased recurrence—including late events beyond five years. By contrast, lesions confined to a single cisternal compartment were more frequently resected completely, demonstrated fewer complications, and remained stable at long-term follow-up. This work represents a 16-year mixed prospective-retrospective cohort, and its findings should be interpreted as associations, not as validation of a predictive model.

Several methodological strengths support the robustness of these results. First, the sample size is relatively large in comparison with existing series. Second, all patients were treated at a single tertiary center, ensuring relative uniformity in surgical strategy, perioperative care, and follow-up protocols. This homogeneity reduces confounding by institutional variability and facilitates consistent comparison across subgroups. Third, the median follow-up exceeded a decade, enabling the detection of late recurrences, which were notable in the multicompartmental and midline-paramedian groups. Inter-rater agreement for the proposed dual-axis classification was strong, supporting its reproducibility in practice. Outcomes were captured not only as overall recovery but also as specific neurological sequelae such as cranial nerve deficits, cerebellar dysfunction, and seizure development. Taken together, these strengths enhance the reliability of the observed associations.

Placed in the context of prior work, the present results refine existing interpretations. Earlier literature on congenital intracranial cysts has traditionally emphasized histology as the principal determinant of recurrence and surgical outcome. Epidermoid cysts, for example, were long regarded as inherently prone to regrowth, often attributed to residual epithelial remnants after resection. The correlation we observed between histology and cisternal localization—particularly the predominance of epidermoid cysts in complex coronal and median-paramedian axial sites—was expected given their embryological tendency toward multicompartmental extension. However, our multivariable analyses demonstrated that once localization was accounted for, histological subtype no longer retained prognostic significance, whereas cisternal configuration remained independently associated with recurrence (complex coronal: OR = 8.09, *p *= 0.001; median/paramedian axial: OR = 5.62, *p* = 0.004). This suggests that part of the “aggressiveness” previously attributed to epidermoids may reflect their frequent distribution across multicompartmental cisterns. Nevertheless, histology remains crucial in surgical planning and patient consent, as certain subtypes (particularly epidermoid and dermoid cysts) are more prone to firm adhesion to neurovascular structures, influencing operative complexity, extent of resection, and long-term surveillance. Thus, the dual-axis cisternal classification should be regarded as a complementary prognostic framework, refining but not replacing the indispensable role of histopathology [3, 13, 15, 17, 19, 21, 24].

Similarly, prior studies have sometimes proposed cyst size as an adverse factor, with larger lesions assumed to carry higher recurrence and morbidity. Our supplementary analyses further explored the role of cyst size as a potential confounding factor. Although maximum dimensions varied significantly across histological subtypes, size did not differ between recurrent and non-recurrent cases and did not alter the predictive value of cisternal localization in multivariable models [12, 15, 18, 20]. Notably, small lesions traversing multiple cisternal planes often proved more challenging to resect completely and demonstrated higher recurrence rates than larger but well-confined cysts. These findings help clarify how histology, size, and anatomical configuration interact.

In terms of surgical strategy, our dual-axis framework provides a structured way to describe surgical decision-making, by outlining how cisternal configuration is associated with feasibility and extent of resection. MCL generally required more cautions strategies, often subtotal removal with staged or combined approaches—such as retrosigmoid followed by subtemporal approach. Dorsal cisternal lesions, median or paramedian, aligned with natural microscopical corridors like retrosigmoid, supracerebellar-infratentorial, or occipital-transtentorial routes, which facilitated complete and safe removal. Ventral lesions showed a distinct dichotomy: ventral paramedian cysts were usually accessible through anterior transpetrosal or subtemporal approaches, whereas ventral median cysts, particularly at the interpeduncular or suprasellar cisterns, presented the greatest technical constraints and necessitated narrow, deep routes such as endonasal or transsphenoidal access. Non-cisternal midline lesions, including colloid and Rathke cleft cysts, were consistently amenable to minimally invasive management, reflecting their unique anatomical setting.

Our analysis shows that nearly one-quarter of complex lesions relapsed beyond five years (23.7% vs. 2.8%, *p* < 0.001), highlighting the need for extended surveillance in this subgroup. Cisternal configuration was closely linked with recurrence: lesions confined to dorsal or ventral paramedian compartments had low recurrence and were usually resectable through standard microsurgical routes, whereas ventral median lesions carried a substantially higher risk, reflecting the constraints of narrow skull base approaches and their proximity to critical structures. MCL showed the greatest recurrence burden, including late events, and often required combined or staged resections. By contrast, non-cisternal midline cysts such as colloid and Rathke’s cleft cysts exhibited low recurrence, consistent with their distinct anatomical profile.

A particularly high-risk subset emerged among patients whose lesions exhibited both multicompartmental extension along the coronal (dorsal–ventral) axis and combined median-paramedian involvement on the axial (medial–lateral) plane. Representing 16.4% of the cohort, this group demonstrated a strikingly elevated recurrence rate of 55.6%, compared to just 14.1% among all other topographic patterns (*p* < 0.001), with an odds ratio of 7.60 (95% CI: 2.53–22.80). This convergence of unfavorable topographic traits highlights the additive risk associated with intersecting cisternal involvement across multiple anatomical planes. From a surgical standpoint, these lesions likely represent the most anatomically challenging configurations—extending across deep midline cisterns while simultaneously encroaching on lateral or dorsal territories—often necessitating combined or staged approaches and increasing the likelihood of subtotal resection. Taken together, these observations emphasize that cisternal topography provides a reproducible and clinically relevant framework for operative planning, anticipating recurrence risk, and guiding the duration of postoperative follow-up.

In terms of outcomes, most patients achieved favorable recovery, consistent with earlier reports on congenital cystic lesions. Patients with multicompartmental lesions had significantly lower recovery, and similar trends were observed for axial median-paramedian lesions. Beyond global outcomes, these subgroups showed a greater incidence of focal deficits—especially cranial nerve VII/VIII dysfunction and cerebellar signs.

The implications for practice emerge in two areas. Preoperatively, cisternal configuration can be used to frame counseling and risk–benefit discussions, providing a clearer basis for setting expectations. Postoperatively, the observed recurrence patterns suggest that surveillance intensity may be adjusted according to anatomical complexity, with multicompartmental and midline-paramedian lesions requiring longer monitoring than confined lesions. At the surgical level, the association between complex topography and higher morbidity highlights the importance of balancing the extent of resection with preservation of neurological function.

Figure 1 present a stepwise algorithm for clinical use of the dual-axis grid: (a) map the cyst on high-resolution MRI; (b) stratify risk based on compartment extension; (c) select the corridor that aligns with its dominant axis; (d) tailor imaging follow-up to the corresponding risk tier.

### Limitations and future directions

The present study consolidates and extends our prior institutional reports, integrating both previously published and newly accrued cases into a single harmonized cohort. Unlike earlier series that addressed specific histological entities or formed part of a systematic review, the current analysis applies a dual-axis cisternal classification across the full spectrum of congenital cystic lesions with longer follow-up and multivariable modeling to examine associations with surgical complexity and recurrence.

Despite the prospective integration and robust statistical analysis, this study is limited by its single-center design, which may constrain external validity. Institutional surgical philosophies, technical expertise, and imaging protocols may vary across centers, potentially affecting the generalizability of the proposed classification system. Furthermore, the heterogeneity in follow-up duration (ranging from 1 to 16 years) raises the possibility of time-to-event imbalance—potentially underestimating late recurrences in recently treated patients and inflating recurrence rates in earlier cohorts. Thus, future multicenter studies with diverse demographic and institutional inputs are necessary to validate the cisternal topographic framework across broader clinical contexts.

Cyst size may also represent a potential confounder. Although maximum dimensions differed significantly across histological subtypes, supplementary analyses indicated that size was not independently associated with recurrence once cisternal localization was considered. Histological distribution was uneven, with epidermoid cysts comprising more than half of the series, while dermoid, neurenteric, and colloid cysts were comparatively underrepresented. This imbalance limits the strength of subgroup analyses and reinforces that the observed patterns represent associations rather than validated predictors.A further limitation is the absence of volumetric lesion assessment and morphometric analysis. Future investigations should integrate cisternal classification with lesion volume, brainstem compression angle, and fiber tract displacement metrics. Multicenter prospective validation is essential—particularly in pediatric populations and rarer cystic subtypes. Incorporating advanced neuroimaging techniques such as Diffusion Tensor Imaging (DTI) and functional MRI may also refine preoperative topographic modeling and risk prediction. Future research should also incorporate standardized patient-reported outcome measures and neurocognitive testing to capture dimensions of quality of life not addressed by current scales.

## Conclusions

While granular case-by-case analysis of histology, surgical approach, and outcome remains indispensable, our dual-axis cisternal model offers a structured framework for risk stratification; nonetheless, its results should be regarded as associative rather than constituting validation of a predictive model. Lesions with multicompartmental extension or involvement of median-paramedian cisterns demonstrated significantly higher rates of subtotal resection, postoperative complications, and delayed recurrence, underscoring the need for extended imaging surveillance and strategic surgical planning.

The proposed dual-axis anatomical framework does not replace histological or size-based classifications but complements them, offering a clinically useful layer of stratification that helps contextualize outcome variability in a more reproductible and transparent manner.

## Supplementary Information

Below is the link to the electronic supplementary material.

ESM 1 Supplementary Material 1 (DOCX 63.8 KB)

## Data Availability

The datasets analyzed during the current study are available from the corresponding author on reasonable request.

## Abbreviations

CSF: Cerebrospinal fluid
AP: Anteroposterior Axis
LL: Laterolateral Axis
CC: Craniocaudal Axis
GTR: Gross total resection
STR: Subtotal resection
MRI: Magnetic Resonance Imaging
CT scan: Computerized Tomography scan
OR: Odds ratio
CI: Confidence Interval
IQR: Interquartile Range
EVD: External Ventricular Drain
VPS: Ventriculo-Peritoneal Shunt
GOS: Glasgow Outcome Scale
MCL: Multicompartmental lesions
CN: Cranial Nerve
SCIT: Supracerebellar Infratentorial
FLAIR: Fluid-Attenuated Inversion Recovery
DTI: Diffusion Tensor Imaging
