# Supplementary material for: A dual-axis cisternal classification for congenital intracranial cystic lesions: implications for surgical strategy and long-term prognosis
Source: Acta Neurochir (Wien). 2025 Dec 1;167(1):309. doi: 10.1007/s00701-025-06722-1 (PMC12672678; doi:10.1007/s00701-025-06722-1)
Supplement: Supplementary file 1 — Supplementary Material 1 (DOCX 63.8 KB) [file 701_2025_6722_MOESM1_ESM.docx]

***Impact of cyst size on recurrence***

***Comparison by histology***

**Supplementary Table 1.** Comparison of maximum dimension in all three investigated axes according to histopathology

| **Histopathology/AP** | **Mean** ± **SD** | **Median (IQR)** | **Mean Rank** | **p*** |
| --- | --- | --- | --- | --- |
| **Colloid** | 21.52 ± 7.1 | 22.5 (16.5-25) | 37.75 | **<0.001** |
| **Dermoid** | 29 ± 13.73 | 32 (19.5-38.5) | 58.12 |  |
| **Epidermoid** | 32.52 ± 11.48 | 29.5 (24.5-39.5) | 64.12 |  |
| **Neurenteric** | 36.13 ± 18.51 | 42 (28.7-46.5) | 65.67 |  |
| **Rathke Cleft** | 15.48 ± 4.34 | 15 (13.8-18) | 18.86 |  |
| **Histopathology/LL** | **Mean** ± **SD** | **Median (IQR)** | **Mean Rank** | **p**** |
| **Colloid** | 22.4 ± 6.6 | 24.5 (17.45-28) | - | **<0.001** |
| **Dermoid** | 34.75 ± 11.52 | 35.5 (27-42.5) | - |  |
| **Epidermoid** | 29.93 ± 10.44 | 28.1 (23-37.5) | - |  |
| **Neurenteric** | 36.46 ± 17.35 | 38 (28.2-45.5) | - |  |
| **Rathke Cleft** | 16.01 ± 2.93 | 16 (14-18) | - |  |
| **Histopathology/CC** | **Mean** ± **SD** | **Median (IQR)** | **Mean Rank** | **p**** |
| **Colloid** | 22.24 ± 7.49 | 22 (16-29) | - | **<0.001** |
| **Dermoid** | 28.5 ± 11.09 | 28.5 (21-36) | - |  |
| **Epidermoid** | 34.45 ± 12.05 | 33.5 (24.4-42.5) | - |  |
| **Neurenteric** | 51.36 ± 18.98 | 50 (41.5-60.5) | - |  |
| **Rathke Cleft** | 15.5 ± 4.05 | 16.1 (12-19) | - |  |

***Kruskal-Wallis H Test, **Welch ANOVA Test**

**
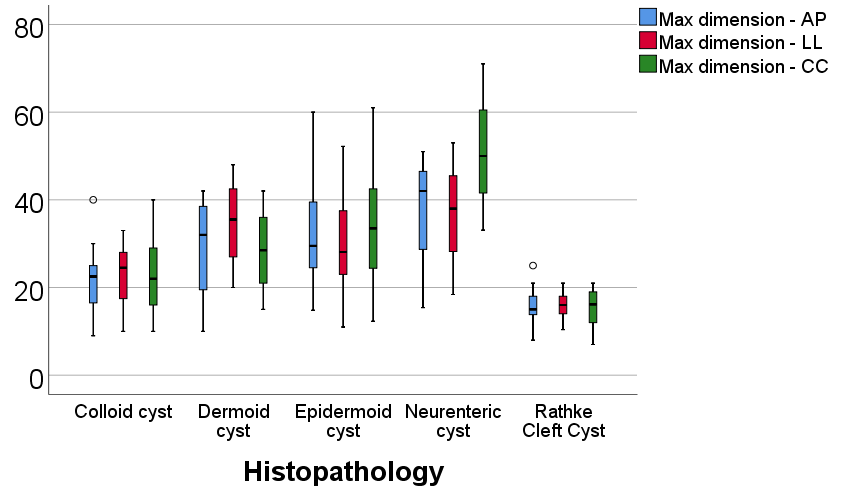
**

**Figure 1.** Comparison of maximum dimension in all three investigated axes according to histopathology

***Comparison by recurrence***

**Supplementary Table 2. Comparison of maximum dimensions on investigated axes according to the existence of recurrence**

| **Dimension axis/Recurrence (Median (IQR))** | **Absent** | **Present** | **p*** |
| --- | --- | --- | --- |
| **Antero-Posterior** | 24 (16-30) | 29.5 (21-39.75) | 0.087 |
| **Latero-lateral** | 24 (17.1-31) | 27 (19.25-37) | 0.248 |
| **Cranio-caudal** | 24 (17.1-36.1) | 30.5 (20-42.75) | 0.154 |

***Mann-Whitney U Test**

**
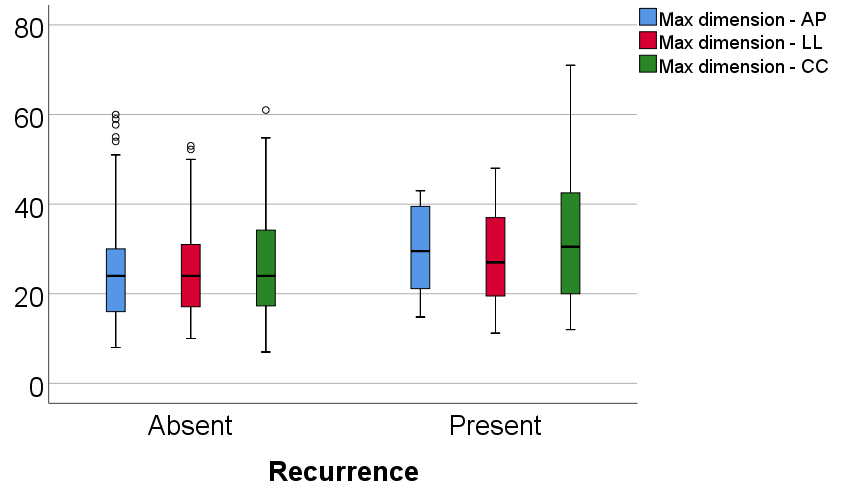
**

**Figure 2. Comparison of maximum dimensions on investigated axes according to the existence of recurrence**

***Multivariable regression***

**Supplementary Table 3. Multivariable binomial logistic regression models used in the prediction of recurrence using localization on coronal/axial axis and maximum antero-posterior dimension**

| **Parameter/Model*** | **OR (95% C.I.)** | **p** |
| --- | --- | --- |
| **Complex – Coronal** | 6.906 (2.149-22.190) | **0.001** |
| **Max dimension - AP** | 0.996 (0.950-1.044) | 0.862 |
| **Parameter/Model**** | **OR (95% C.I.)** | **p** |
| **Median&Paramedian – Axial** | 5.702 (1.646-19.759) | **0.006** |
| **Max dimension – AP** | 0.997 (0.951-1.045) | 0.891 |

****Multivariable binomial logistic regression model, χ² (2) = 13.053, p=0.001, Nagelkerke R2 = 0.197, Hosmer and Lemeshow Test, p=0.617, Sensitivity = 100%; Specificity = 0%, Overall accuracy =79.4%***

*****Multivariable binomial logistic regression model, χ² (2) = 8.986, p=0.011, Nagelkerke R2 = 0.139, Hosmer and Lemeshow Test, p=0.371, Sensitivity = 100%; Specificity = 0%, Overall accuracy =79.4%***

***Relative prognostic contribution of histology vs. cisternal localization***

**Supplementary Table 4.** Distribution of the patients according to histopathology and type of cysts according to coronal/axial axis localization

| ***Histopathology/***  ***Coronal axis*** | **Other** | **Complex** | | **p*** |
| --- | --- | --- | --- | --- |
|  | **%** | **%** |  | |
| **Colloid** | **36.1%** | **0%** | **<0.001** | |
| **Dermoid** | 2.8% | 7.9% |  |  |
| **Epidermoid** | **34.7%** | **84.2%** |  |  |
| **Neurenteric** | 1.4% | 7.9% |  |  |
| **Rathke Cleft** | **25%** | **0%** |  |  |
| ***Histopathology/***  ***Axial axis*** | **Other** | **Median&Paramedian** | | **p*** |
|  | **%** | **%** |  | |
| **Colloid** | **28.9%** | **0%** | **<0.001** | |
| **Dermoid** | 3.3% | 10% |  |  |
| **Epidermoid** | **43.3%** | **90%** |  |  |
| **Neurenteric** | 4.4% | 0% |  |  |
| **Rathke Cleft** | **20%** | **0%** |  |  |

***Fisher’s Exact Test**

**Supplementary Table 5**. Univariable and multivariable binomial logistic regression models used in the prediction of recurrence based on coronal/axial localization and histopathology

| **Parameter** | **Univariable** | | **Multivariable*** | |
| --- | --- | --- | --- | --- |
|  | **OR (95% C.I.)** | **p** | **OR (95% C.I.)** | **p** |
| **Complex location (Coronal)** | 6.753 (2.457-18.565) | **<0.001** | 8.094 (2.394-27.37) | **0.001** |
| **Epidermoid cysts** | 2.009 (0.773-5.224) | 0.152 | 0.707 (0.207-2.417) | 0.581 |
| **Parameter** | **Univariable** | | **Multivariable**** | |
|  | **OR (95% C.I.)** | **p** | **OR (95% C.I.)** | **p** |
| **Median&paramedian location (Axial)** | 5.923 (2.062-17.016) | **0.001** | 5.621 (1.756-17.989) | **0.004** |
| **Epidermoid cysts** | 2.009 (0.773-5.224) | 0.152 | 1.122 (0.375-3.353) | 0.837 |

***Multivariable enter approach model, χ² (2) = 15.461, p<0.001, Nagelkerke R^2^ = 0.204, Hosmer and Lemeshow Test, p =0.426, Sensitivity = 100%, Specificity = 0%, Overall accuracy = 79.1%**

****Multivariable enter approach model, χ² (2) = 10.790, p=0.005, Nagelkerke R^2^ = 0.146, Hosmer and Lemeshow Test, p =0.526, Sensitivity = 88.5%, Specificity = 34.8%, Overall accuracy = 77.3%**

**Supplementary Table 6.** Distribution of the patients according to the existence of recurrence and histopathology type

| ***Recurrence/***  ***Type*** | **Absent** | **Present** | **p*** |
| --- | --- | --- | --- |
|  | **%** | **%** |  |
| **Colloid** | 27.6% | 8.7% | 0.062 |
| **Dermoid** | 3.4% | 8.7% |  |
| **Epidermoid** | 48.3% | 65.2% |  |
| **Neurenteric** | 2.3% | 8.7% |  |
| **Rathke Cleft** | 18.4% | 8.7% |  |
| ***Recurrence/***  ***Epidermoid type*** | **Absent** | **Present** | **p*** |
|  | **%** | **%** |  |
| **Other type** | 51.7% | 34.8% | 0.167 |
| **Epidermoid** | 48.3% | 65.2% |  |

***Fisher’s Exact Test**

**Supplementary Table 7.** Univariable and multivariable binomial logistic regression models used in the prediction of recurrence based on coronal/axial localization and histopathology

| **Parameter** | **Multivariable*** | |
| --- | --- | --- |
|  | **OR (95% C.I.)** | **p** |
| **Complex location (Coronal)** | 5.925 (1.509-23.267) | **0.011** |
| ***Histopathology (Reference = Rathke Cleft)*** | - | - |
| **Colloid** | 0.667 (0.085-5.228) | 0.700 |
| **Dermoid** | 1.663 (0.121-22.906) | 0.704 |
| **Epidermoid** | 0.871 (0.128-5.923) | 0.888 |
| **Neurenteric** | 2.021 (0.124-32.815) | 0.621 |
| **Parameter** | **Multivariable**** | |
|  | **OR (95% C.I.)** | **p** |
| **Median&paramedian location (Axial)** | 4.961 (1.497-16.434) | **0.009** |
| ***Histopathology (Reference = Rathke Cleft)*** | - | - |
| **Colloid** | 0.667 (0.085-5.228) | 0.700 |
| **Dermoid** | 2.676 (0.223-32.127) | 0.438 |
| **Epidermoid** | 1.526 (0.281-8.282) | 0.624 |
| **Neurenteric** | 8.000 (0.690-92.703) | 0.096 |

***Multivariable enter approach model, χ² (2) = 16.227, p<0.001, Nagelkerke R^2^ = 0.214, Hosmer and Lemeshow Test, p =0.903, Sensitivity = 97.7%, Specificity = 17.4%, Overall accuracy = 80.9%**

****Multivariable enter approach model, χ² (2) = 15.313, p=0.009, Nagelkerke R^2^ = 0.203, Hosmer and Lemeshow Test, p =0.806, Sensitivity = 97.7%, Specificity = 17.4%, Overall accuracy = 80.9%**

Overall, it can be safely assumed that the localization of cysts based on coronal/axial axes remain a significant and independent predictor over recurrence, even when adjusting for histopathology.
